# Loss of Hepatocyte-Nuclear-Factor-1α Impacts on Adult Mouse Intestinal Epithelial Cell Growth and Cell Lineages Differentiation

**DOI:** 10.1371/journal.pone.0012378

**Published:** 2010-08-24

**Authors:** Carine R. Lussier, François Brial, Sébastien A. B. Roy, Marie-Josée Langlois, Elena F. Verdu, Nathalie Rivard, Nathalie Perreault, François Boudreau

**Affiliations:** 1 Département d'Anatomie et Biologie Cellulaire, Faculté de Médecine et des Sciences de la Santé, Université de Sherbrooke, Sherbrooke, Quebec, Canada; 2 Division of Gastroenterology, McMaster University, Hamilton, Ontario, Canada; Charité-Universitätsmedizin Berlin, Germany

## Abstract

**Background and Aims:**

Although Hnf1α is crucial for pancreas and liver functions, it is believed to play a limited functional role for intestinal epithelial functions. The aim of this study was to assess the consequences of abrogating Hnf1α on the maintenance of adult small intestinal epithelial functions.

**Methodology/Principal Findings:**

An *Hnf1α* knockout mouse model was used. Assessment of histological abnormalities, crypt epithelial cell proliferation, epithelial barrier, glucose transport and signalling pathways were measured in these animals. Changes in global gene expression were also analyzed. Mice lacking *Hnf1α* displayed increased crypt proliferation and intestinalomegaly as well as a disturbance of intestinal epithelial cell lineages production during adult life. This phenotype was associated with a decrease of the mucosal barrier function and lumen-to-blood glucose delivery. The mammalian target of rapamycin (mTOR) signalling pathway was found to be overly activated in the small intestine of adult *Hnf1α* mutant mice. The intestinal epithelium of *Hnf1α* null mice displayed a reduction of the enteroendocrine cell population. An impact was also observed on proper Paneth cell differentiation with abnormalities in the granule exocytosis pathway.

**Conclusions/Significance:**

Together, these results unravel a functional role for Hnf1α in regulating adult intestinal growth and sustaining the functions of intestinal epithelial cell lineages.

## Introduction

The small intestinal epithelium consists of villi and crypts lined with proliferative stem and progenitor cells. These cells ensure constant renewal of the epithelium throughout the life of the individual [1], [2]. The suckling-weaning transition occurs during the third post-natal week and leads to profound changes in intestinal gene expression and epithelial cell function for the establishment of the adult functional mucosa [3], [4]. The mature epithelium of the small intestine is composed of four major cell lineages including enterocytes, enteroendocrine, goblet and Paneth cells. Enterocytes express several intestinal epithelium gene products involved in carbohydrates and lipids absorption, transport and metabolism as well as in barrier integrity to ensure protection from the luminal side and optimal blood nutrient and hormone delivery to the organism.

Several transcription factors have been identified to participate in intestinal epithelial cell determination and gut functions [1], [4]. The zinc finger GATA4 is crucial for jejunal absorption of dietary fat [5]. Conditional ablation of the homeobox *Cdx2* in the mouse intestinal endoderm impairs intestinal identity [6]. The transcriptional regulators *Hnf1α* and *Hnf4α* are direct transcriptional targets of Cdx2 in this context [6]. Loss of *Hnf4α* during late mouse embryonic gut development results in minor alterations of intestinal homeostasis [7]. The impact of sole *Hnf1α* deletion is currently assumed to be of no consequence on adult intestinal epithelium homeostasis [8], [9]. Bosse and colleagues reported that Hnf1α was required for lactase-phlorizin hydrolase gene expression *in vivo* but did not investigate the overall impact of Hnf1α loss on the intestinal transcriptome and intestinal epithelial functions [10]. Very recently, D'Angelo and colleagues reported that both Hnf1α and Hnf1β were required to be inactivated in order to impact on intestinal epithelial terminal differentiation in mice [9]. In the course of their studies, these authors only focused on young animals and did not provide with an extensive characterization of the *Hnf1α* null mouse intestinal phenotype.

HNF1α is a homeodomain-containing transcription factor expressed in the liver, kidney, pancreas, stomach and intestine [11], [12]. *Hnf1α* knockout mouse models display both Laron-type dwarfism and non-insulin-dependent diabetes characterized also with a marked liver enlargement [13], [14] and alterations in the maintenance of normal endocrine pancreas [15]. Hnf1α is expressed in the nucleus of intestinal epithelial cells in the embryo and early after birth and vanishes during the first two weeks of mouse post-natal life to reappear during the suckling-weaning transition[8] [16]. These observations are in line with the possibility that intestinal Hnf1α expression could be of no functional importance during post-natal development and become relevant during the onset and the maintenance of adult life.

To clarify the impact of Hnf1α loss of function on intestinal epithelial physiology, we undertook the characterization of adult small intestinal epithelium integrity in a mouse model that was knocked out for the *Hnf1α* gene [14]. We report that loss of Hnf1α results in important intestinal epithelial disorders affecting enterocyte barrier function, enterocyte glucose transport, homeostasis of crypt proliferation and disturbance of intestinal epithelial cell lineages functions during adult life.

## Results

### *Hnf1α* deletion alters the architecture of adult small intestinal epithelium and leads to intestinalomegaly

**Figure 1:**
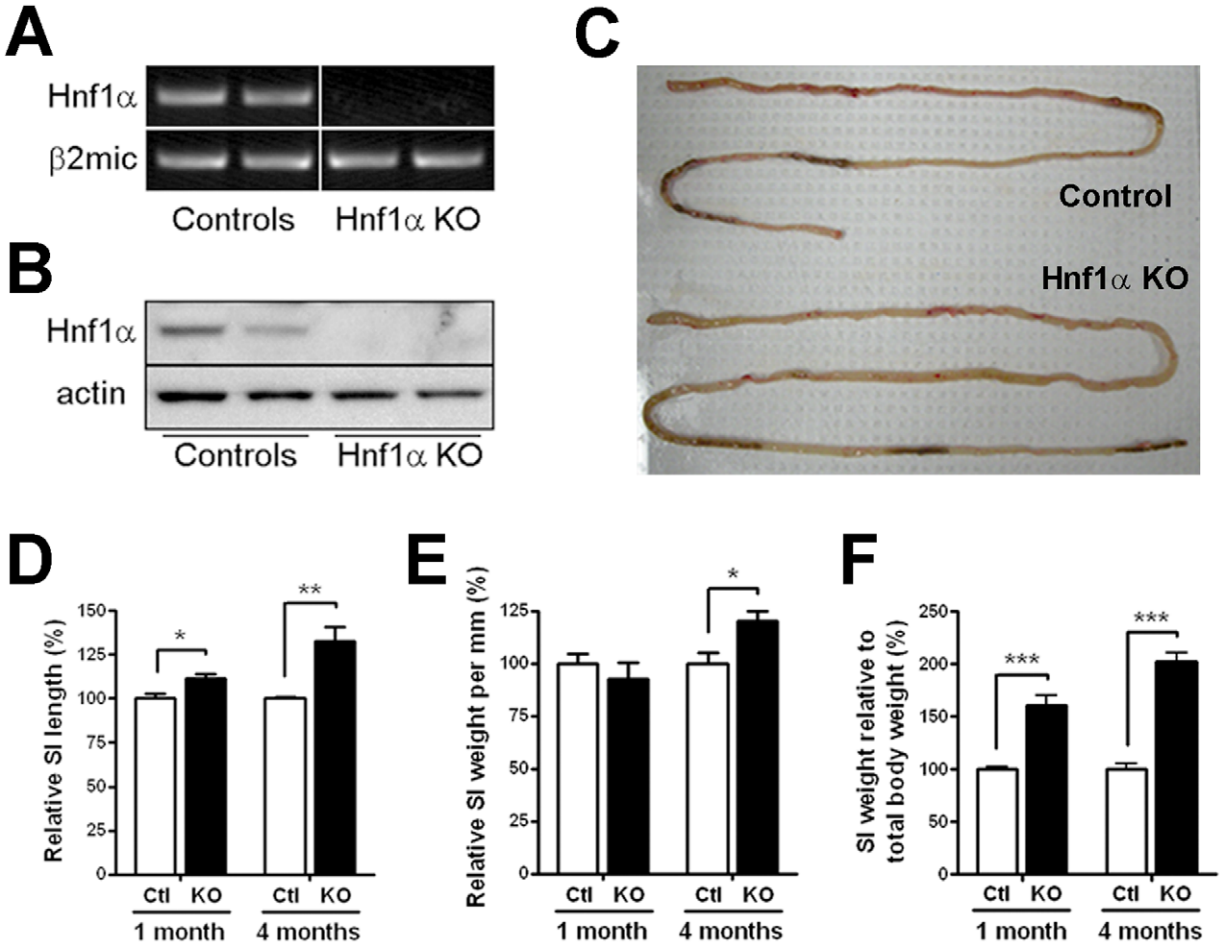
*Hnf1α* null mice display intestinalomegaly. (A) RT-PCR detection of *Hnf1α* was performed on total small intestinal RNA extracts. *β2-microglobulin* (β2mic) mRNA level was monitored as a housekeeping gene control. (B) Western blot analysis was performed on total small intestinal mucosa lysates with an antibody specific for the detection of Hnf1α. Specific detection of actin was done to control for protein integrity. (C) Photograph of representative 5-month-old control and *Hnf1α* null small intestines. Statistical analysis of 4-month-old mice small intestine (SI) relative length (D), relative weight per mm (E) and weight relative to mouse total body weight (*F*). n = 5-10; *P<0.05; **P<0.01;***P<0.001.

**Figure 2:**
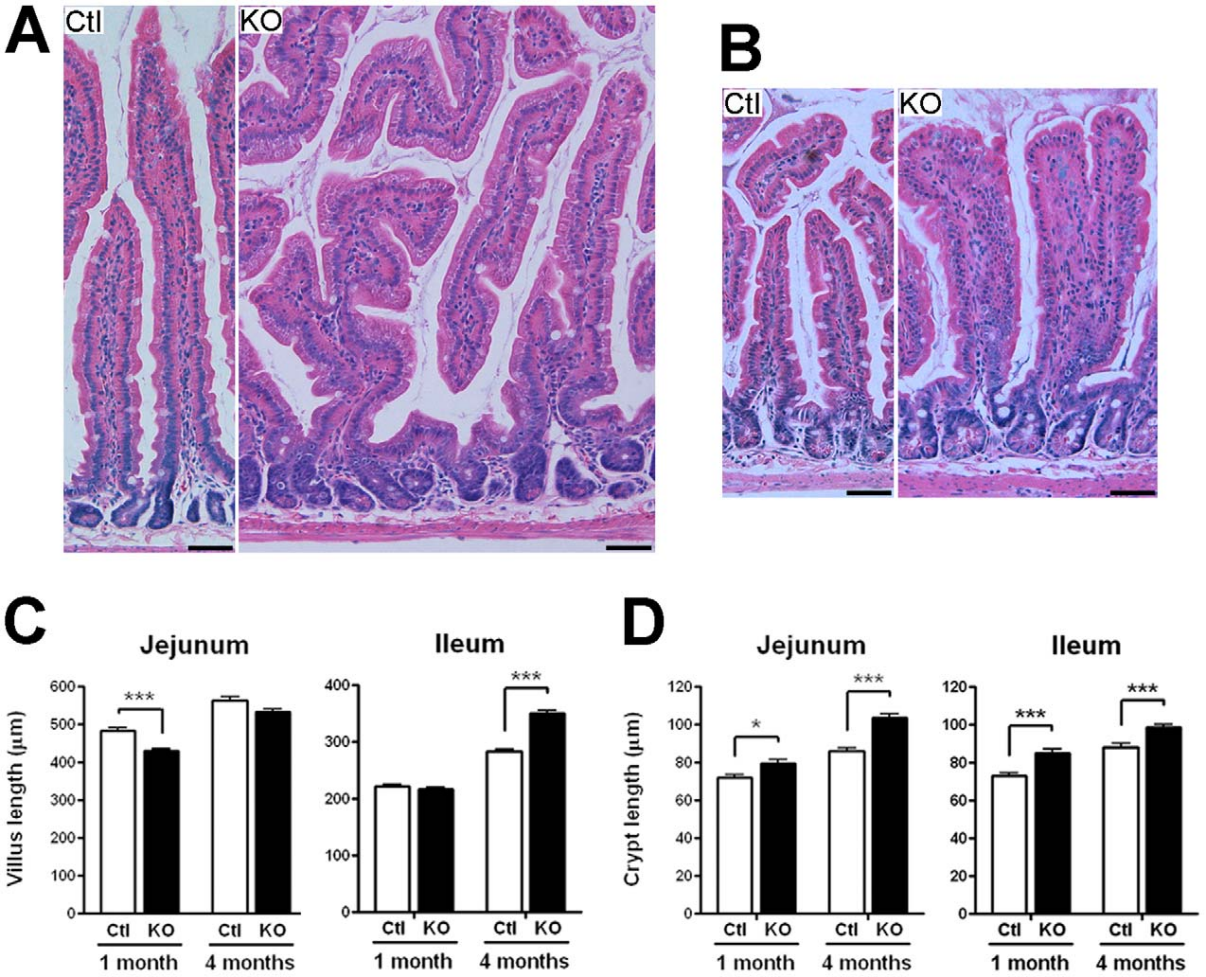
Loss of *Hnf1α* results in disturbed crypt-villus architecture. Haematoxylin and eosin stained micrographs of the jejunum (A) and the ileum (B) of 4-month-old mice. Bar = 50 µm. Statistical analysis of villus (C) and crypt length (D) of jejunum and ileum of 1-month and 4-month-old mice. n = 3; total of 99-120 villi and 120 crypts. *P<0.05; ***P<0.001.

**Figure 3:**
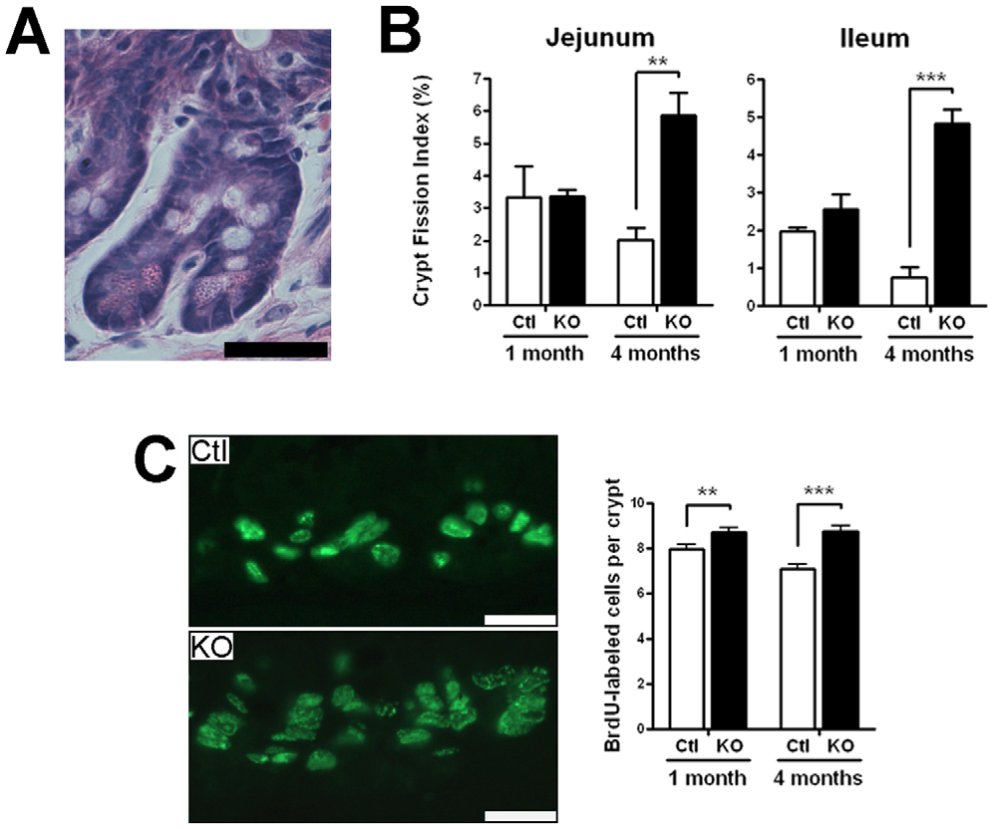
Loss of *Hnf1α* results in increased crypt proliferation index. (A) Haematoxylin and eosin stained micrograph of a representative dividing crypt event. Bar = 30 µm. (B) Statistical analysis of the percentage of dividing crypt unit (crypt proliferation index) in the jejunum and ileum of 1-month and 4-month-old mice. n = 3-4. (C) Representative immunofluorescence for BrdU performed on sections of jejunum of both control (Ctl) and *Hnf1α* null mice (KO) (left panels). A statistical analysis of the number of BrdU labeled cells in the jejunum of 1-month and 4-month-old mice. n = 3; total of 85 and 105 crypts, respectively (right panel). **P<0.01; ***P<0.001.

A mouse *Hnf1α* knockout colony was generated by breeding the previously reported *Hnf1α* mutant strain in which *Hnf1α* gene expression was inactivated with the removal of the first exon that resulted in the abolition of *Hnf1α* gene transcription [14]. As previously reported, *Hnf1α* mutant mice displayed a critical reduction in their growth during post-natal development that was maintained during adulthood (data not shown). We observed that ∼25% of the mutant died during the first 36 h after birth. Total RNA and protein isolated from the jejunum and subjected to RT-PCR and Western showed that mutant mice did not produce significant levels of wild-type *Hnf1α* mRNA (Figure 1A) and protein (Figure 1B). Macroscopic analysis of intestinal morphology in young *Hnf1α* mutant pups did not reveal significant difference with control littermates. However, the intestine of adult *Hnf1α* mutant animals was longer and markedly expanded (Figure 1C) despite the fact these mutant mice were significantly reduced in size as compared with those of control littermates. Statistical analysis indicated a significant increase of 1.33-fold in length (Figure 1D), 1.20-fold in relative weight per length (Figure 1E) and 2.02-fold in relative weight to total body weight (Figure 1F) of the small intestines of 4 month-old mutant mice compared with those of control littermates. Significant but less prominent increases in the length and relative weight of the small intestine were also observed in 1 month old mutant animals as compared with those of control littermates (Figure 1D and F). Hematoxylin and eosin staining of jejunum and ileum sections revealed abnormal branching of the villi in the mutant animals (Figure 2A and B, right panels), a feature that was first observed at 1 month-old to become more frequent in the adult intestinal mucosa. A significant 11% decrease of jejunum villi length was observed in 1 month-old *Hnf1α* mutant mice as compared to control mice (Figure 2C, left panel). In opposition, a significant 23.6% increase of ileum villi length was observed in 4 month-old *Hnf1α* mutant mice as compared to control mice (Figure 2C, right panel). Jejunum and ileum crypts length was significantly increased (10.2% at 1 month and 20.3% at 4 months for the jejunum; 16.5% at 1 month and 11.8% at 4 months for the ileum) in *Hnf1α* mutant mice as compared to control mice (Figure 2D). Increase in intestinal crypt proliferation, a process of crypt fission that occurs more frequently during the first 3 weeks of post-natal life to decline afterward [17], was also observed in adult mutant mice compared with those of control littermates. The intestinal crypt fission rate, calculated as the number of dividing crypts per 200 crypts per animal (Figure 3A), was significantly up-regulated more than 2.9-fold in the jejunum and 6.5-fold in the ileum (Figure 3B) in adult *Hnf1α* mutant mice as compared to controls. This rate did not reach significance in 1-month-old *Hnf1α* mutant mice (Figure 3B). A minor but significant 10% increase of the index of epithelial cell proliferation was observed in the small intestine of 1-month-old *Hnf1α* mutant mice as determined by BrdU incorporation (Figure 3C). This tendency was more marked (23%) in 4-month-old *Hnf1α* mutant mice (Figure 3C).

### The intestinal epithelial mTOR/S6K signalling pathway, but not the β-catenin pathway, is upregulated in adult Hnf1α deleted mice

**Figure 4:**
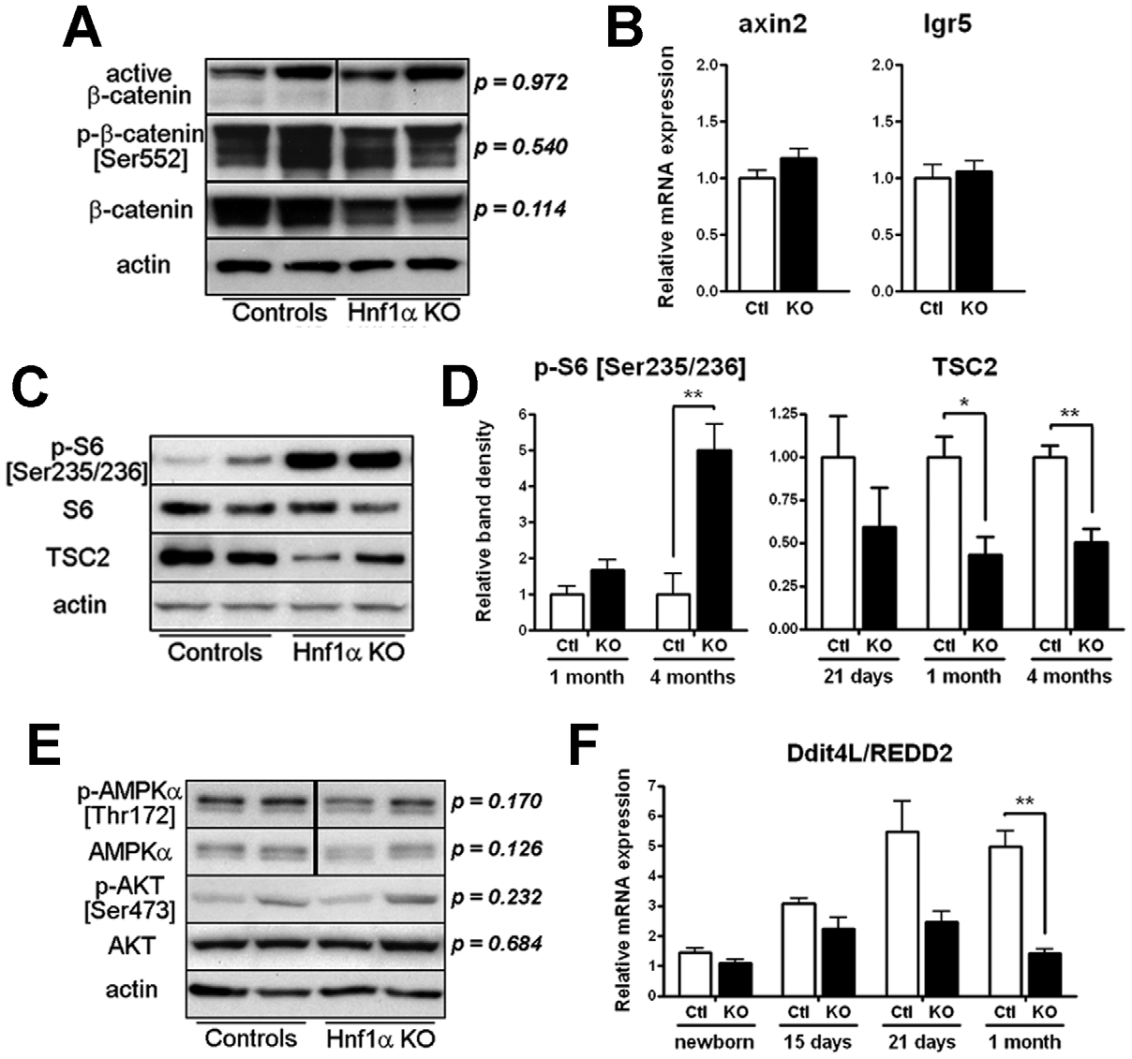
*Hnf1α* null mice display an increase in mTOR signalling. (A) Total protein extracts of 4-month-old mice were analyzed by Western blot for the detection of the active form of β-catenin dephosphorylated on Ser37 or Thr41, the active form of β-catenin phosphorylated on Ser552 and the total β-catenin. Band densities relative to the actin level were calculated for each sample and expression variation was statistically validated. n = 4. (B) qRT-PCR analysis of axin2 and lgr5 was performed on total RNA extracts from 4-month-old mice. n = 5-10. (C) Total protein extracts of 4-month-old mice were analyzed by Western blot for the detection of S6 ribosomal protein phosphorylated on serine 235/236, total S6 and total tuberous sclerosis 2 (TSC2). Specific detection of actin was done to control for protein integrity. (D) Total protein extracts were analyzed by Western blot for the detection of S6 ribosomal protein phosphorylated on serine 235/236 and total TSC2. Band densities relative to the actin level were calculated for each sample and expression variation was statistically validated. n = 3-6. (E) Total protein extracts of 4-month-old mice were analyzed by Western blot for the detection of total and activated forms of AMPKα and AKT. Band densities relative to the actin level were calculated for each sample and expression variation was statistically validated. n = 4-7. (F) qRT-PCR analysis of Ddit4L/Redd2 was performed on total RNA extracts at different time points during post-natal development. n = 5-10 per time point. *P<0.05; **P<0.01.

Adult *Hnf1α* mutant mice displayed a dramatic increase in intestinal crypt proliferation resulting in organomegaly. Because the Wnt/β-catenin signaling pathway is crucial in the regulation of gut epithelial cell proliferation [1], the level of β-catenin activity was next compared between *Hnf1α* mutant and control mice. Western blot analysis on total extracts obtained from small intestinal mucosa scrapping showed no significant modulation of total β-catenin between *Hnf1α* mutant and control mice (Figure 4A). No change in the level of unphosphorylated active form of β-catenin on serine 37 was observed. We have also verified the phosphorylation status of β-catenin on serine 552, a process regulated by AKT and reported to promote β-catenin stability and nuclear localization [18]. The phosphorylation status of β-catenin at this specific amino acid residue remained unchanged between *Hnf1α* mutant and controls (Figure 4A). This finding was also confirmed by immunofluorescence for which no difference in nuclear β-catenin labelling was observed (data not shown). The expression of the *Axin2* and stem cells expressing leucine rich repeat-containing G protein-coupled receptor 5 (*Lgr5*) gene transcripts, two well established targets of Wnt signalling in intestinal epithelial cells [19], [20], was not affected by the loss of *Hnf1α* (Figure 4B). One specific signalling pathway that regulates cell growth and metabolism is mTOR [21]. To investigate the activation status of the mTOR signalling pathway, the phosphorylation of S6, which is catalyzed by S6 kinase in an mTOR-dependent manner [22], was monitored in the small intestinal epithelium of *Hnf1α* mutant and controls mice. Western blot analysis showed that the S6 phosphorylation at Ser235/236 was elevated more than 5-fold in the small intestinal epithelium of 4 month-old *Hnf1α* mutant mice as compared with control mice (Figure 4C–D). S6 phosphorylation levels was not yet significantly modulated in the small intestine of 1 month-old *Hnf1α* mutant mice (Figure 4D) which was consistent with the observed delay in the amplification of crypt unit proliferation during the adult life of these mutant mice (Figure 3). One crucial negative regulator of mTOR signalling is the tuberous sclerosis protein TSC2 [21]. Although TSC2 gene transcript remained stable during post-natal development and adult life (data not shown), TSC2 protein was down modulated by 50% in the small intestinal epithelium of adult *Hnf1α* mutant mice as compared with control mice (Figure 4C–D). The PI3K/Akt and AMP-activated protein kinase (AMPK) pathways are important regulators of TSC2 protein function and stability. AMPK and AKT phosphorylation-activated status was not significantly modulated in the small intestinal epithelium of 4-month-old *Hnf1α* mutant mice as compared with control mice under our experimental conditions (Figure 4E). Another important molecular entity that inhibits mTOR signalling is the Ddit4l/REDD2 that acts downstream of Akt and upstream of TSC2 preventing the inhibitory binding of 14-3-3 to TSC2 [23]. Since no commercial antibodies were available for Ddit4l/REDD2, total RNA was isolated from the small intestine of *Hnf1α* mutant and control mice at different time points during post-natal development. qRT-PCR analyses showed that the Ddit4l/REDD2 gene transcript was significantly increased during the suckling-weaning transition in control mice, a tendency that was abolished in the small intestine of *Hnf1α* null mice (Figure 4F).

### The enterocyte differentiation program, mucosal barrier function and lumen-to-blood glucose delivery are altered in Hnf1α deleted mice

**Figure 5:**
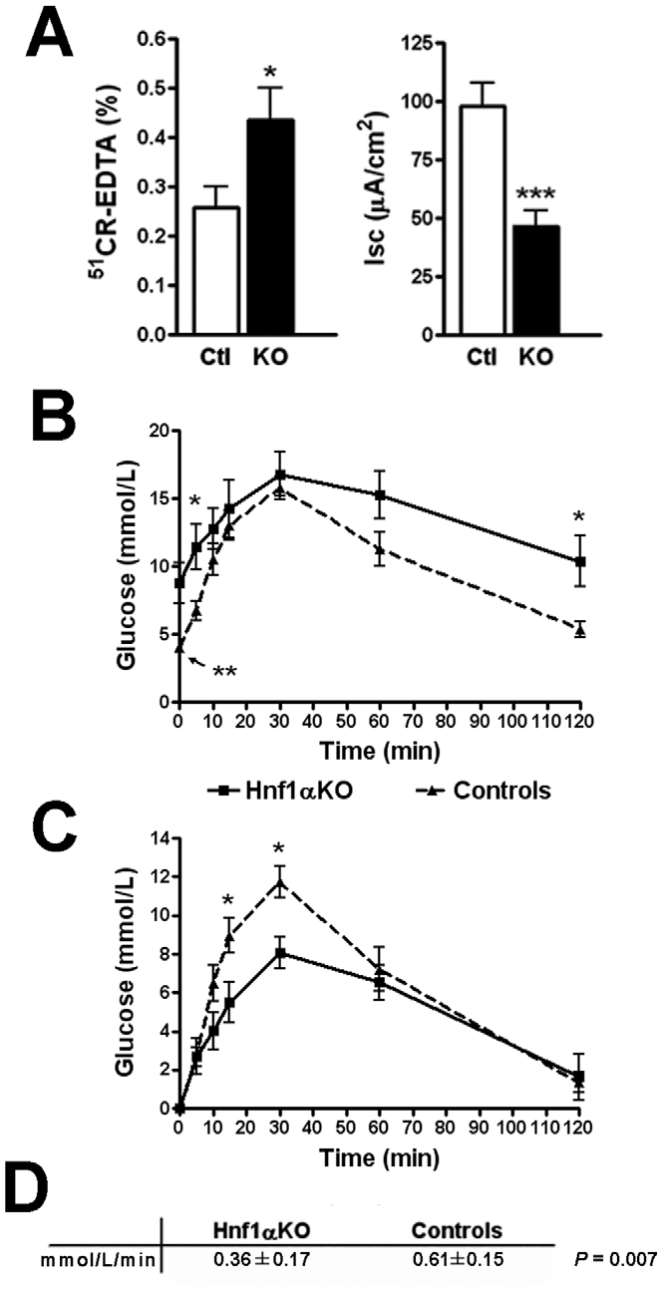
Barrier functions and glucose transport are deregulated in the *Hnf1α* mutant intestine. (A) Statistical analysis of small intestinal permeability for ^51^CR-EDTA and ion transport as measured by short circuit current (*I*_sc_). n = 5-6. (B) Glucose plasma concentration at 0 (before) and 5, 10, 15, 30, 60, and 120 min after administration of glucose through gastric gavage. n = 6-8. (C) Glucose plasma concentration net increase after calibration of starting glucose concentration for each group at 0. (D) Linear regression T test analysis of glucose plasma concentration between 0 and 15 min. *P<0.05; ***P<0.001.

A gene expression profiling was next performed to investigate whether the loss of *Hnf1α* could have an impact on the intestinal gene network and epithelial associated functions. A statistical analysis (*p*≤0.05) predicted 317 unique mouse transcripts to be modulated between adult control and mutant jejunum samples (differential ratio ≥2.0, Table S1). To gain insight into how these modifications relate to epithelial cell functions, IPA software was utilized to categorize the down-modulated genes by biological function. This analysis identified carbohydrate metabolism, small molecule biochemistry and transport, lipid metabolism and endocrine system development as being the most important gene networks predicted to be down-modulated in the *Hnf1α* mutant small intestine (Table S2). Intestinal barrier function was next assessed by Ussing chamber technique since several genes involved in ion transport and barrier function were shown to be significantly altered in *Hnf1α* mutant. Adult *Hnf1α* mutant mice showed a significant 69% increase in small intestinal paracellular permeability to ^51^Cr-EDTA as compared to controls (Figure 5A, left panel). In addition, baseline *I*_sc_ was decreased more than 52% in the small intestine of adult *Hnf1α* null mice as compared to controls demonstrating that a decrease in active epithelial ion transport was also taking place in these animals (Figure 5A, right panel). Decrease in intestinal mucosal ion transport can impact on lumen-to-blood glucose transport [24]. In addition, *Hnf1α* null mice displayed an important down-regulation of G6PC and glucose-6-phosphate transporters (Table S1), both direct targets for Hnf1α transcriptional action [25], [26]. In order to test whether intestinal epithelial cells were affected in their capacity to deliver blood glucose from intestinal luminal content, we undertook standardized oral administration of D-glucose in overnight fasted *Hnf1α* mutant and control mice. As previously reported [15], the basal blood glucose concentration was significantly higher in *Hnf1α* mutant as compared to control mice (Figure 5B). Blood glucose levels peaked at 15–30 min after glucose administration and thereafter declined in both *Hnf1α* mutant and control mice (Figure 5B). However, the rate of plasma glucose delivery during the first 15 min following gastric glucose administration was significantly lowered by more than 43.3% in *Hnf1α* mutant when compared to control mice (Figure 5C–D).

### The Paneth and enteroendocrine cell lineages are deregulated in Hnf1α deleted mice

**Figure 6:**
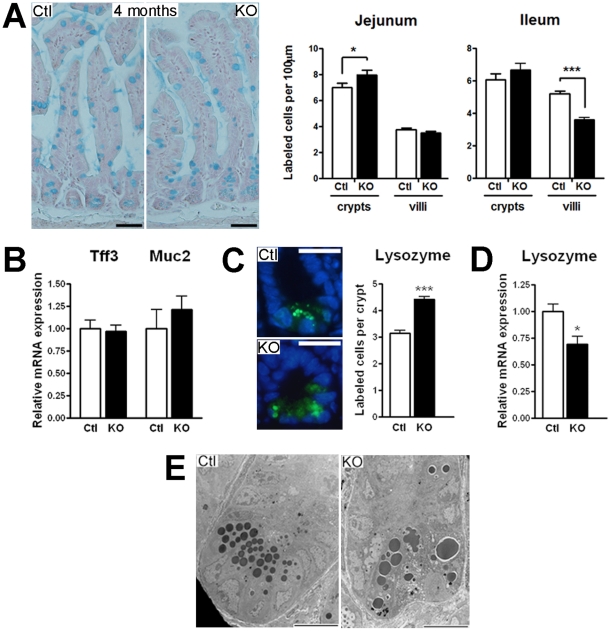
Loss of *Hnf1α* leads to deregulation of Paneth cells homeostasis. (A) Jejunum and ileum tissue sections were used to perform alcian blue staining. Representative micrographs and statistical analyses of the number of positive labeled-cells per length units of crypts and villi. n = 3; total of 75 villi and 60 crypts. Bar = 50 µm (B) Quantitative RT-PCR (qRT-PCR) analysis of trefoil factor 3 (Tff3) and mucin 2 (Muc2) was performed on total RNA extracts from 4-month-old mice. n = 5-10. (C) Lysozyme immunodetection was performed on ileum tissue sections of 4-month-old mice. Nuclei were stained in blue with Dapi. Representative micrographs and statistical analyses of the number of positive cells per crypt. n = 3-4; average of 35 crypts per animal. Bar = 20 µm (D) qRT-PCR analysis of lysozyme was performed on total RNA extracts from 4-month-old mice. n = 5-10. (E) Electron microscopic analysis of 4-month-old mice ileum sections. Bar = 10 µm. *P<0.05; ***P<0.001.

**Figure 7:**
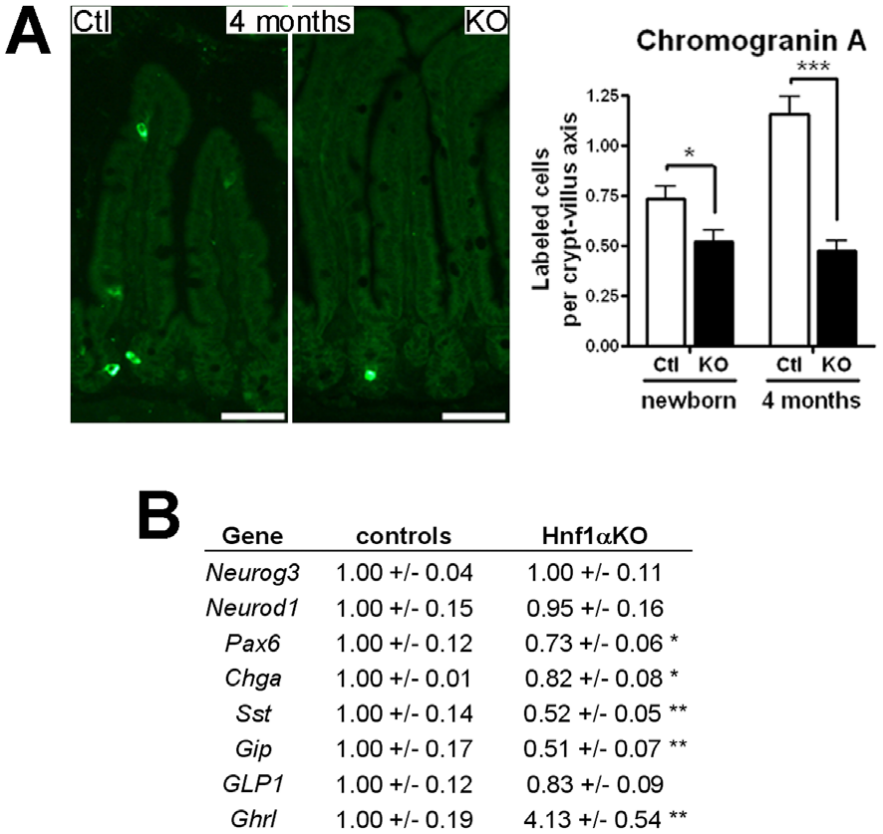
Loss of *Hnf1α* leads to deregulation of enteroendocrine cell production. (A) Chromogranin A immunodetection was performed on ileum tissue sections. Representative micrographs and statistical analyses of the number of positive labeled-cells per crypt-villus axis. n = 3-4; average of 40 crypt-villus axis per mouse. (B) qRT-PCR analysis performed on total RNA extracts from 4-month-old mice (means +/- SEM). n = 5-10. *P<0.05; **P<0.01; ***P<0.001.

The effect of *Hnf1α* deletion on the maturation of intestinal epithelial secretory cell lineages was next monitored. The number of goblet cells was first compared between *Hnf1α* mutant and control mice by alcian blue staining. The relative number of goblet cells per length units was modestly but significantly increased in the jejunum crypts of *Hnf1α* mutant mice and significantly decreased in the ileum villi of the same individuals (Figure 6A). However, these subtle modifications did not impact on overall gene transcript expression of goblet cell markers as revealed by qRT-PCR analyses of trefoil factor 3 and mucin 2 transcripts expression (Figure 6B). Although lysozyme staining of the Paneth cells was consistently more diffused in *Hnf1α* mutant crypts, a significant 40.5% increase in the number of positive cells per crypt was denoted in the *Hnf1α* mutant mice as compared to control mice (Figure 6C, right panel). qRT-PCR analyses showed a significant 30.6% reduction in the expression of lysozyme transcript in the *Hnf1α* mutant mice as compared to control mice (Figure 6D). Electron micrograph of Paneth cells confirmed abnormal cellular morphology with aberrant and disorganized granules in the *Hnf1α* mutant mice (Figure 6E). Thus, the number of Paneth cells was increased but these cells were less mature in *Hnf1α* mutant mice. The number of chromogranin A positive enteroendocrine cells per villus was significantly decreased in newborn (29%) and adult (59%) mutant mice as compared to control mice (Figure 7A). The analysis of several targets associated with enteroendocrine differentiation revealed significant modulations of chromogranin A (Chga), somatostatin (Sst), gastric inhibitory polypeptide (Gip), Ghrelin (Ghrl) and Pax6 gene transcripts expression (Figure 7B). Overall, these observations indicated that loss of *Hnf1α* had a significant impact on terminal differentiation of multiple intestinal epithelial cell lineages.

## Discussion

Although Hnf1α was demonstrated to regulate several intestinal epithelial specific genes *in vitro*, the general assumption was that Hnf1α loss of function did not significantly influence intestinal homeostasis [9] [10]. Our analysis demonstrates a fundamental role for Hnf1α in regulating adult intestinal epithelium functions including enterocyte barrier and metabolic functions, enteroendocrine cell specification and Paneth cell maturation. Recent findings suggest that *Hnf1α* and *Hnf1β* were both needed to be inactivated in order to significantly affect intestinal epithelial functions [9]. We provide here the important conclusion that abrogation of *Hnf1α* alone is important to maintain intestinal epithelium basic functions during adulthood. One possible explanation for our divergent conclusions is that these authors restrained their analyses to young adult mice, a period where the defects noted in our analysis are in the process of being manifested.

We provide two possibilities to explain the long time-frame intestinal effects associated with the loss of Hnf1α function. First, the delayed intestinal phenotype could be associated with the profile of Hnf1α protein distribution during intestinal post-natal development. Indeed, Hnf1α protein expression is shut down during early intestinal post-natal development to become reexpressed at the suckling-weaning transition [10], [16]. Thus, the effect of removing Hnf1α expression would not affect intestinal functions during this specific developmental transition because of natural decline of Hnf1α intestinal epithelial expression in control mice. Coincidently, the expression pattern for TSC2 and Ddit4l/REDD2 are affected in Hnf1α null mice beginning at this transition. During post-natal intestinal growth, most new crypts will emerge from a process of crypt fission that is highly active in a short postnatal period to decline drastically after weaning [27]. Thus, Hnf1α spontaneous down-modulation during early post-natal intestinal development could result in a molecular cascade that no longer restricts intestinal crypt proliferation and tissue growth, a process that is overly persisting in adult *Hnf1α* mutant mice and thus becoming only apparent on the long term. One important component that governs intestinal crypt proliferation is the APC/β-catenin pathway [17]. Loss of *Hnf1α* function did not influence intestinal epithelial β-catenin activity based on β-catenin phosphorylation status, nuclear localization and expression assessments of direct β-catenin dependent transcriptional targets. The mTOR signalling pathway, whose activation has been functionally linked to the development of many cancers including colonic adenomas [28], was found to be up-regulated in *Hnf1α* null intestinal epithelium and could be responsible for the amplification of crypt proliferation and intestinal growth. A second possibility to explain the long term nature of the intestinal defects as observed in this mouse model could be related to non-autonomous causes dependent on other Hnf1α depleted tissues. Future experimental designs to specifically and solely delete the *Hnf1α* gene in the intestinal epithelium will address this matter.

We have recently reported that deletion of intestinal epithelial *Pten* led to intestinalomegaly [29], an observation that was consistent to what seen in many other organs [30]. In addition, branching of villi and multiplication of crypts units were similarly observed in absence of epithelial Pten [29]. It has been well described that Pten, through inactivation of Akt, can negatively control translation mechanisms through its regulation of mTOR/S6K signalling, thus influencing cell growth and protein synthesis [22]. Our data highlight the sustained activation of the mTOR signalling pathway in the intestinal epithelium of *Hnf1α* mutant mice correlating with the activation of a subset of genes involved in translational regulation (Table S3). However, the mTOR interacting components influenced by the loss of Hnf1α are more likely to be independent of Pten signalling since Akt remained unchanged in the intestinal epithelium of *Hnf1α* null and control mice. AMPK, an energy sensor kinase that directly phosphorylates TSC2 to promote its activity for inhibition of mTOR [31], was not significantly altered in the intestinal mucosa of *Hnf1α* mutant mice under our experimental conditions. Ddit4l/REDD2 induction of expression was observed in the small intestine during the suckling-weaning transition in normal mice, a process that was inhibited in *Hnf1α* null mice. However, it remains unclear whether Ddit4l/REDD2 is directly targeted by Hnf1α during this transition. This possible molecular interaction represents a perspective for future directions.

Similar analogies can be made between our observations and the megaintestine phenotype that was reported to occur in claudin-15 deficient mice [32]. The lack of claudin-15 was shown to decrease paracellular ion conductance in gut epithelial cells resulting in hyperproliferation [32], fluid accumulation and the formation of multiple lumens in the zebrafish gut [33]. Although claudin-15 gene transcript was not found to be modulated in the jejunum of *Hnf1α* null mice (Table S1), the findings that electric conductance and several solute carrier transporter genes were significantly impaired in the intestine of *Hnf1α* null mice are common to the claudin-15 null mice phenotype and thus could contribute to the increase in epithelial cell proliferation and the manifestation of the megaintestine phenotype as observed in our mouse model.

Total body energy homeostasis is importantly challenged in *Hnf1α* mutant mice [34] [13] [15]. We found an important number of direct Hnf1α gene targets related to carbohydrate and lipid metabolism to be down modulated in the intestinal epithelium of adult *Hnf1α* mutant mice. The small intestine is the primary organ for lipids and glucose uptake, nutrients processing and then delivery to the circulating system. A defect in the expression of several lipids and glucose transporters as well as intestinal lumen-to-blood glucose delivery from epithelial cells could suggest an alteration in nutrient absorption that might also contribute to the wasting syndrome driven by the loss of *Hnf1α* [13].

Loss of *Hnf1α* impacts on the production of chromogranin A positive cells already at birth implicating a novel and direct role for this regulator in the differentiation of this cell lineage. Since Neurogenin 3 and NeuroD1 expression was not significantly modulated in the intestine of *Hnf1α* mutant mice, Hnf1α must act downstream of Neurogenin 3 and furthermore, independently of NeuroD1, to influence differentiation of chromogranin A cell subtypes. Paneth cells are crucial entities to protect the gut epithelium from bacterial invasions. A strong phenotypic parallel can be made with altered Paneth cell maturation that we observed in *Hnf1α* null mice and Paneth cell defect that occurs in mice deficient for the autophagy gene *Atg16l1* [35], [36]. ATG16L1-deficient Paneth cells exhibited abnormalities in the granule exocytosis pathway, a feature that was common to Crohn's disease patients that were homozygous for the *ATG16L1* disease risk allele. ATG1 kinase plays a pivotal role in the positive control of autophagy [37]. mTOR activation blocks ATG1 kinase activity resulting in inhibition of autophagy [38]. These phenotypic similarities suggest that Hnf1α could be functionally linked to molecular components that participate to the regulation of autophagy.

In conclusion, we have reported that Hnf1α loss of function impacts on adult intestinal epithelial cell basic functions in mice. We also showed that Hnf1α was potent to regulate intestinal epithelium growth in restricting activation of the mTOR signalling pathway specifically during the post-weaning period. These observations imply a critical and unsuspected role for Hnf1α in adult intestinal epithelium maintenance.

## Materials and Methods

### Animals and Ethics statement

The *Hnf1α* knockout mouse model was described elsewhere [14]. Mice were kept under pathogen free conditions and were tested negative for helicobacter, pasteurella and murine norovirus. *Hnf1α* knockout and control littermates were treated in accordance with a protocol reviewed and approved by the Institutional Animal Research Review Committee of the Université de Sherbrooke (approval ID number 102-10B) in conformity with the Canadian Council on Animal Care.

### Histologic staining and immunofluorescence

Small intestinal segments were fixed in 4% paraformaldehyde overnight at 4°C, dehydrated, embedded in paraffin and cut to 5-µm sections. Alcian blue and hematoxylin and eosin staining, as well as immunofluorescence were performed exactly as previously described [7]. The following affinity-purified antibodies were used: mouse anti-BrdU-fluorescein (11202693001; Roche Applied Science), rabbit Lysozyme (N1515; Dako), rabbit chromogranin A (20085; Immunostar) and rabbit Ki67 (RM-9106-R7; Thermo scientific).

### Ion transport and permeability measurement

Each jejunum segment of mouse aged of 3 months was opened along the mesenteric border, rinsed, and mounted in an Ussing chamber. Epithelial ion transport and permeability measurements were done exactly as described before [39].

### Gastric gavage and plasma glucose measurement

D-glucose diluted in water was administered to overnight fasted 2-month-old mice mice through a gastric tube (20G×1-1/2"; Popper and sons). Glucose plasma level was measured immediately before gastric gavage and at times 5, 10, 15, 30, 60 and 120 min with a FreeStyle Light apparatus (Abbott).

### RNA isolation and RT-PCR

Total RNA from small intestinal segment was isolated and qRT-PCR performed exactly as previously described [40]. Results were individually calibrated with TATA box binding protein (TBP). Primer sequences are available upon request.

### Microarray screening and data analysis

Probes for microarray analysis were generated from RNA isolated from the distal jejunum of 3 control and 3 *Hnf1α* ko mice at 4 months of age. Affymetrix GeneChip® Mouse Genome 430 2.0 arrays were screened via the microarray platform of McGill University and Génome Québec Innovation Center as previously described [41] (data are accessible through GEO series accession number GSE23040 and are all MIAME compliant). To test for statistically significant changes in signal intensity (*p* values of ≤0.05; EB Wright & Simon), compiled data (RMA analysis) were screened using FlexArray 1.1 (Genome Quebec). Data were analyzed through the use of Ingenuity Pathways Analysis (IPA) to generate networks of genes associated with biological functions and/or diseases (Ingenuity Systems, www.ingenuity.com).

### Western blot analysis

Enrichment of mucosal total protein extracts were obtained from starved mice ileum segments as previously reported [29] and electrophoresis and blotting were performed exactly as previously described [42]. The following antibodies from Santa Cruz Biotechnology were used: goat HNF1α (SC-6547), goat HNF4α (SC-6556), goat actin (SC-1615), rabbit phospho-AMPKα Thr172 (SC-33524). The following antibodies from Cell Signaling were used: rabbit phospho-β-catenin Ser552 (9566), rabbit S6 ribosomal protein (2217), rabbit phospho-S6 ribosomal protein Ser235/236 (4858), rabbit Tuberin/TSC2 (3612), rabbit phospho-Tuberin/TSC2 Thr1462 (3617), rabbit Hamartin/TSC1 (4906), rabbit AMPKα (2532), rabbit AKT (9272), rabbit phospho-Akt Ser473 (9271). These antibodies were also used: mouse active-β-catenin dephosphorylated on Ser37 or Thr41 (05-665; Millipore), mouse β-catenin (610154; BD Biosciences), rabbit CDX2/3 (CDX2/3-NR, [43]).

### Quantification and statistical analysis

Morphometry was determined using the MetaMorph software (Universal Imaging). Statistical analyses were performed using the GraphPad Prism 4 software. Statistics were calculated using the two-way Student's two-tailed *t* test, two-way nested analysis of variance (ANOVA), or by the Mann-Whitney *U* test. Differences were considered significant with a *P* value of <0.05.

## Supporting Information

Table S1 Genes that display a 2-fold modulation of their expression in Hnf1α mutant vs control jejunum (P value less than or equal to 0.05, N = 3).
(0.10 MB PDF)

Table S2 Genes annotated by IPA as involved in intestinal epithelial functions with altered expression in Hnf1α mutant jejunum samples as compared with control jejunum samples (N = 3).
(0.03 MB PDF)

Table S3 Genes annotated by IPA as involved in protein translation control with modified expression in Hnf1α mutant jejunum samples as compared with control jejunum samples (N = 3).
(0.03 MB PDF)
