# Supplementary material for: Loss of Hepatocyte-Nuclear-Factor-1α Impacts on Adult Mouse Intestinal Epithelial Cell Growth and Cell Lineages Differentiation
Source: PLoS One. 2010 Aug 24;5(8):e12378. doi: 10.1371/journal.pone.0012378 (PMC2927538; doi:10.1371/journal.pone.0012378)
Supplement: Table S1 — Genes that display a 2-fold modulation of their expression in Hnf1α mutant vs control jejunum (P value less than or equal to 0.05, N = 3). (0.10 MB PDF) [file pone.0012378.s001.pdf]

Supplemental Table 1. Genes that display a 2-fold modulation of their expression in *Hnf1α* mutant vs control jejunum (P value ≤0.05, N=3)

Up-regulated

Gene Title	Gene Symbol	Fold change	P-value
2'-5' oligoadenylate synthetase-like 1	Oasl1	2.19	4.47E-05
abhydrolase domain containing 3	Abhd3	2.45	1.47E-04
actin, alpha 1, skeletal muscle	Acta1	2.15	5.43E-03
acyl-CoA thioesterase 12	Acot12	2.53	8.77E-06
adenylosuccinate lyase 1	Adsl	2.08	2.83E-03
aldo-keto reductase family 1, member B8	Akr1b8	4.99	1.27E-04
aldo-keto reductase family 1, member C14	Akr1c14	3.13	5.43E-03
aldolase 3, C isoform	Aldoc	2.72	6.73E-06
amphiregulin	Areg	4.10	1.46E-02
ankyrin repeat domain 37	Ankrd37	2.91	1.84E-03
ankyrin repeat domain 56	Ankrd56	2.75	9.29E-05
arginase 1, liver	Arg1	2.01	4.28E-02
Arrestin domain containing 3	Arrdc3	3.01	4.35E-02
asparagine synthetase	Asns	2.45	3.51E-04
ATP-binding cassette, sub-family A (ABC1), member 1	Abca1	2.14	5.93E-04
ATP-binding cassette, sub-family B (MDR/TAP), member 1A	Abcb1a	4.34	6.68E-06
ATP-binding cassette, sub-family C (CFTR/MRP), member 3	Abcc3	2.74	7.72E-06
camello-like 5	Cml5	3.56	1.21E-04
carbonic anhydrase 13	Car13	2.89	2.91E-04
carboxylesterase 6	Ces6	2.99	3.83E-05
CD38 antigen	Cd38	4.18	6.38E-08
chemokine (C-C motif) ligand 20	Ccl20	2.29	3.57E-02
chemokine (C-C motif) ligand 28	Ccl28	2.80	2.73E-05
cysteine-rich hydrophobic domain 1	Chic1	2.42	4.99E-03

cytochrome P450, family 2, subfamily a, polypeptide 4	Cyp2a4	6.03	1.39E-02
cytochrome P450, family 2, subfamily d, polypeptide 10	Cyp2d10	3.25	3.30E-03
cytochrome P450, family 2, subfamily j, polypeptide 13	Cyp2j13	2.20	9.10E-03
cytochrome P450, family 2, subfamily u, polypeptide 1	Cyp2u1	2.15	2.29E-04
cytochrome P450, family 3, subfamily a, polypeptide 13	Cyp3a13	2.84	9.64E-07
cytochrome P450, family 4, subfamily f, polypeptide 16	Cyp4f16	3.62	2.81E-04
cytochrome P450, family 51	Cyp51	2.77	8.71E-05
D site albumin promoter binding protein	Dbp	2.20	1.08E-04
D-amino acid oxidase 1	Dao1	3.12	6.17E-04
DEAD (Asp-Glu-Ala-Asp) box polypeptide 56	Ddx56	2.49	1.23E-05
defensin related cryptdin, related sequence 12	Defcr-rs12	6.05	2.20E-03
defensin related cryptdin, related sequence 2	Defcr-rs2	4.59	2.19E-02
defensin related cryptdin, related sequence 7	Defcr-rs7	5.45	2.13E-02
dentin matrix protein 1	Dmp1	2.66	1.40E-02
diacylglycerol kinase, delta	Dgkd	2.05	1.48E-03
DNA segment, Chr 17, human D6S56E 5	D17H6S56E-5	2.49	7.67E-04
DNA-damage-inducible transcript 4	Ddit4	2.30	5.88E-04
DnaJ (Hsp40) homolog, subfamily B, member 3	Dnajb3	2.05	4.81E-03
dynamin 1-like	Dnm1l	2.48	1.94E-02
EGL nine homolog 3 (C. elegans)	Egln3	2.15	3.77E-03
endothelial differentiation, lysophosphatidic acid G-protein-coupled receptor 7	Edg7	3.68	1.00E-05
epoxide hydrolase 1, microsomal	Ephx1	2.46	6.86E-03
farnesyl diphosphate synthetase	Fdps	2.07	5.95E-03
fatty acid binding protein 6, ileal (gastrotropin)	Fabp6	6.54	1.21E-02
fibronectin leucine rich transmembrane protein 2	Flrt2	4.07	8.28E-04
forkhead box A1	Foxa1	2.03	1.46E-05
G protein-coupled receptor 108	Gpr108	2.37	1.54E-06

gastric intrinsic factor	Gif	5.76	5.43E-04
ghrelin	Ghrl	3.93	3.83E-05
glia maturation factor, beta	Gmfb	2.08	2.92E-03
glucosaminyl (N-acetyl) transferase 3, mucin type	Gcnt3	2.24	1.87E-06
glutathione S-transferase, alpha 1 (Ya)	Gsta1	4.09	5.46E-03
glutathione S-transferase, alpha 4	Gsta4	2.81	2.69E-03
glutathione S-transferase, mu 3	Gstm3	2.80	4.44E-04
haloacid dehalogenase-like hydrolase domain containing 3	Hdhd3	3.02	1.62E-04
heat shock protein 1A	Hspa1a	2.77	6.18E-03
heat shock protein 1B	Hspa1b	2.46	1.25E-02
heparan sulfate (glucosamine) 3-O-sulfotransferase 1	Hs3st1	2.41	4.52E-06
histocompatibility 2, Q region locus 10	H2-Q10	2.28	1.99E-02
histone cluster 3, H2a	Hist3h2a	2.29	9.55E-04
HLA-B-associated transcript 1A	Bat1a	3.05	2.48E-03
hyaluronan mediated motility receptor (RHAMM)	Hmmr	2.03	6.52E-04
hydroxysteroid (17-beta) dehydrogenase 13	Hsd17b13	31.16	2.40E-07
Immunoglobulin heavy chain (gamma polypeptide)	Ighg	2.20	9.39E-03
Immunoglobulin kappa chain variable 1 (V1)	Igk-V1	3.31	9.83E-03
isopentenyl-diphosphate delta isomerase	Idi1	2.04	3.87E-03
kalirin, RhoGEF kinase	Kalrn	2.41	2.85E-04
lactamase, beta 2	Lactb2	2.15	2.53E-04
mannosyl (alpha-1,3-)-glycoprotein beta-1,4-N-acetylglucosaminyltransferase, isozyme C (putative)	Mgat4c	4.92	2.91E-04
membrane targeting (tandem) C2 domain containing 1	Mtac2d1	2.35	1.09E-03
methylenetetrahydrofolate dehydrogenase (NAD+ dependent), methenyltetrahydrofolate cyclohydrolase	Mthfd2	2.44	4.72E-03
MOCO sulphurase C-terminal domain containing 1	Mosc1	2.51	2.91E-03
NAD(P)H dehydrogenase, quinone 1	Nqo1	2.34	1.32E-03
open reading frame 9	ORF9	2.35	2.98E-04
otopetrin 3	Otop3	2.95	2.04E-03

paired related homeobox 1	Prrx1	2.01	2.18E-04
palmitoyl-protein thioesterase 1	Ppt1	2.48	1.26E-05
period homolog 1 (Drosophila)	Per1	2.06	1.57E-03
period homolog 2 (Drosophila)	Per2	2.75	2.70E-04
peroxisome proliferator activated receptor gamma	Pparg	2.58	7.61E-03
phospholipase A2, group V	Pla2g5	3.80	7.62E-04
phosphoserine aminotransferase 1	Psat1	3.01	1.11E-03
pipecolic acid oxidase	Pipox	2.72	6.65E-05
pirin	Pir	3.20	3.92E-05
pleckstrin homology domain containing, family H (with MyTH4 domain) member 1	Plekhh1	2.11	2.07E-02
protease, serine, 12 neurotrypsin (motopsin)	Prss12	2.38	1.04E-04
PTK6 protein tyrosine kinase 6	Ptk6	2.42	3.09E-04
RAB37, member of RAS oncogene family	Rab37	3.26	1.95E-03
regenerating islet-derived 3 gamma	Reg3g	2.24	2.84E-03
rhomboid, veinlet-like 2 (Drosophila)	Rhbdl2	2.23	1.83E-03
Rho-related BTB domain containing 3	Rhobtb3	2.90	1.33E-05
ribosomal protein L21	Rpl21	2.07	1.67E-04
serum amyloid A 1	Saa1	2.76	9.99E-05
similar to inward rectifier potassium channel Kir7.1		2.06	7.27E-03
six transmembrane epithelial antigen of prostate 2	Steap2	2.35	1.11E-04
six transmembrane epithelial antigen of the prostate 1	Steap1	4.96	6.78E-06
solute carrier family 16 (monocarboxylic acid transporters), member 6	Slc16a6	2.06	1.08E-02
solute carrier family 19 (sodium/hydrogen exchanger), member 3	Slc19a3	4.65	3.58E-07
solute carrier family 25 (mitochondrial carrier, Aralar), member 12	Slc25a12	2.59	1.02E-05
solute carrier family 7 (cationic amino acid transporter, y+ system), member 1	Slc7a1	2.02	9.67E-04
solute carrier family 7 (cationic amino acid transporter, y+ system), member 5	Slc7a5	2.16	1.15E-03
squalene epoxidase	Sqle	2.69	5.90E-04

suppressor of cytokine signaling 3	Socs3	2.40	5.51E-03
synaptotagmin binding, cytoplasmic RNA interacting protein	Syncrip	2.06	4.77E-05
thioredoxin interacting protein	Txnip	2.65	5.06E-03
transcription factor 23	Tcf23	2.02	9.14E-04
transferrin receptor	Tfrc	3.33	5.65E-04
transmembrane phosphoinositide 3-phosphatase and tensin homolog 2	Tpte2	2.19	1.60E-04
transmembrane protein 116	Tmem116	2.49	6.47E-04
tripartite motif protein 16	Trim16	2.18	1.56E-05
ubiquitin D	Ubd	4.03	4.85E-02
ubiquitin specific peptidase 12	Usp12	2.03	2.09E-03
ubiquitin specific peptidase 2	Usp2	2.56	4.70E-05
WD repeat domain 18	Wdr18	2.57	1.18E-03
X-linked myotubular myopathy gene 1	Mtm1	2.39	3.20E-04
zinc finger protein 33B	Zfp33b	2.55	3.40E-05
zinc finger protein 711	Zfp711	2.03	4.39E-04

Down-regulated

Gene Title	Gene Symbol	Fold change	P-value
2',3'-cyclic nucleotide 3' phosphodiesterase	Cnp	0.46	9.47E-06
3-hydroxy-3-methylglutaryl-Coenzyme A synthase 2	Hmgcs2	0.44	4.60E-03
5' nucleotidase, ecto	Nt5e	0.13	2.25E-04
acyl-CoA synthetase long-chain family member 3	Acsl3	0.48	3.31E-05
adrenergic receptor, alpha 2a	Adra2a	0.46	5.91E-03
alanyl (membrane) aminopeptidase	Anpep	0.49	8.01E-05
aldo-keto reductase family 1, member C12	Akr1c12	0.05	1.17E-07
aldo-keto reductase family 1, member C13	Akr1c13	0.04	2.68E-06
aldo-keto reductase family 1, member C19	Akr1c19	0.13	1.81E-03

alpha fetoprotein	Afp	0.01	5.16E-09
amnionless	Amn	0.12	6.17E-08
amphiphysin	Amph	0.44	3.43E-03
amyloid beta (A4) precursor-like protein 1	Aplp1	0.21	2.93E-04
ankyrin 3, epithelial	Ank3	0.48	9.78E-04
annexin A13	Anxa13	0.30	5.78E-06
anterior gradient homolog 3 (Xenopus laevis)	Agr3	0.43	2.57E-03
apolipoprotein A-IV	Apoa4	0.43	1.29E-03
apolipoprotein C-II	Apoc2	0.24	3.09E-06
aquaporin 1	Aqp1	0.30	3.11E-06
aquaporin 7	Aqp7	0.29	9.37E-06
arginase type II	Arg2	0.43	3.32E-07
arylacetamide deacetylase (esterase)	Aadac	0.05	1.67E-06
ataxin 1	Atxn1	0.21	1.71E-02
ATP-binding cassette, sub-family G (WHITE), member 8	Abcg8	0.28	2.05E-06
B-cell CLL/lymphoma 11A (zinc finger protein)	Bcl11a	0.50	2.99E-02
Bcl-2-related ovarian killer protein	Bok	0.43	4.62E-03
blocked early in transport 1 homolog (S. cerevisiae)	Bet1	0.49	2.48E-06
bone morphogenetic protein 8a	Bmp8a	0.44	3.43E-03
camello-like 1	Cml1	0.36	4.48E-05
carboxypeptidase B2 (plasma)	Cpb2	0.22	2.31E-04
carboxypeptidase M	Cpm	0.44	7.37E-05
catalase	Cat	0.49	2.61E-04
cathepsin C	Ctsc	0.34	1.66E-04
chemokine (C-C motif) ligand 24	Ccl24	0.48	9.31E-05
Chemokine (C-C motif) receptor 9	Ccr9	0.25	4.99E-06
chloride channel calcium activated 3	Clca3	0.46	3.57E-04

chloride intracellular channel 6	Clic6	0.48	1.40E-03
choline kinase alpha	Chka	0.48	1.84E-05
c-mer proto-oncogene tyrosine kinase	Mertk	0.47	4.72E-04
complement component 4 binding protein	C4bp	0.35	3.33E-04
creatine kinase, brain	Ckb	0.24	1.40E-03
crystallin, zeta	Cryz	0.48	1.38E-02
CTD (carboxy-terminal domain, RNA polymerase II, polypeptide A) small phosphatase-like	Ctdspl	0.25	6.60E-06
cubilin (intrinsic factor-cobalamin receptor)	Cubn	0.23	4.15E-04
cytochrome P450, family 2, subfamily c, polypeptide 55	Cyp2c55	0.16	7.63E-06
cytochrome P450, family 2, subfamily c, polypeptide 688	Cyp2c68	0.06	5.60E-08
cytochrome P450, family 2, subfamily d, polypeptide 26	Cyp2d26	0.21	2.59E-05
cytochrome P450, family 3, subfamily a, polypeptide 11	Cyp3a11	0.22	1.43E-03
cytochrome P450, family 3, subfamily a, polypeptide 25	Cyp3a25	0.23	3.37E-05
cytochrome P450, family 3, subfamily a, polypeptide 41A	Cyp3a41a	0.48	1.46E-03
DEP domain containing 6	Depdc6	0.47	1.55E-02
dimethylarginine dimethylaminohydrolase 1	Ddah1	0.32	6.06E-05
dipeptidase 1 (renal)	Dpep1	0.18	2.82E-05
dipeptidylpeptidase 4	Dpp4	0.35	9.15E-06
disabled homolog 1 (Drosophila)	Dab1	0.48	8.37E-05
dopa decarboxylase	Ddc	0.06	1.11E-06
endothelin 1	Edn1	0.36	3.67E-04
esterase 1	Es1	0.46	5.14E-04
estrogen-related receptor gamma	Esrrg	0.45	2.80E-03
eukaryotic translation elongation factor 1 alpha 2	Eef1a2	0.44	1.21E-05
exophilin 5	Exph5	0.34	7.41E-06
fatty acid binding protein 1, liver	Fabp1	0.06	1.68E-07
Fc receptor, IgG, alpha chain transporter	Fcgrt	0.19	2.95E-05

FERM, RhoGEF and pleckstrin domain protein 2	Farp2	0.33	1.57E-06
fibroblast growth factor 1	Fgf1	0.34	1.78E-06
flavin containing monooxygenase 5	Fmo5	0.47	2.84E-04
Fraser syndrome 1 homolog (human)	Fras1	0.28	9.07E-05
G protein-coupled receptor 39	Gpr39	0.44	4.06E-04
gamma-glutamyltransferase 1	Ggt1	0.06	2.58E-05
gap junction membrane channel protein beta 1	Gjb1	0.42	9.13E-06
gene model 566, (NCBI)	Gm566	0.31	2.19E-02
glucose-6-phosphatase, catalytic	G6pc	0.32	1.65E-04
glutamate dehydrogenase 1	Glud1	0.21	2.24E-08
glutathione S-transferase, theta 1	Gstt1	0.30	4.56E-04
glycerol kinase	Gyk	0.48	6.65E-04
golgi integral membrane protein 4	Golim4	0.45	1.16E-04
golgi transport 1 homolog A (S. cerevisiae)	Golt1a	0.11	2.38E-07
growth arrest specific 6	Gas6	0.45	7.88E-03
growth factor receptor bound protein 14	Grb14	0.38	5.78E-03
growth factor receptor bound protein 7	Grb7	0.48	9.44E-06
guanine nucleotide binding protein (G protein), gamma 11	Gng11	0.39	7.11E-06
guanylate cyclase activator 2a (guanylin)	Guca2a	0.09	2.35E-05
guanylate cyclase activator 2b (retina)	Guca2b	0.04	4.47E-07
H2A histone family, member V	H2afv	0.41	7.63E-06
hepatocyte growth factor activator	Hgfac	0.38	1.49E-04
huntingtin interacting protein 1 related	Hip1r	0.41	3.04E-06
hydroxysteroid (17-beta) dehydrogenase 13	Hsd17b13	0.49	3.59E-02
hydroxysteroid (17-beta) dehydrogenase 6	Hsd17b6	0.08	2.40E-09
inositol hexaphosphate kinase 2	Ihpk2	0.47	4.24E-05
insulin-like growth factor binding protein 3	Igfbp3	0.41	7.07E-03

interleukin 15	Il15	0.49	1.05E-04
islet amyloid polypeptide	Iapp	0.42	1.85E-03
lactase	Lct	0.13	1.46E-04
lactate dehydrogenase B	Ldhb	0.44	2.04E-02
leukocyte cell-derived chemotaxin 2	Lect2	0.37	8.05E-04
lipin 2	Lpin2	0.47	1.60E-05
major facilitator superfamily domain containing 2	Mfsd2	0.47	2.72E-04
similar to NADP-dependent malic enzyme (NADP-ME) (Malic enzyme 1)	Mod1	0.47	2.66E-04
mannose binding lectin (C)	Mbl2	0.15	2.35E-03
mast cell protease 1	Mcpt1	0.38	2.00E-02
mast cell protease 2	Mcpt2	0.41	1.01E-02
meprin 1 alpha	Mep1a	0.21	9.13E-06
metaxin 3	Mtx3	0.46	5.20E-06
metaxin 3	Mtx3	0.44	2.12E-04
methyltransferase like 7A	Mettl7a	0.35	3.57E-06
mitochondrial carrier homolog 2 (C. elegans)	Mtch2	0.43	5.11E-03
mucolipin 3	Mcoln3	0.41	1.10E-03
myeloid ecotropic viral integration site-related gene 1	Mrg1	0.48	5.77E-03
myotubularin related protein 11	Mtmr11	0.03	1.96E-10
N-acetylated alpha-linked acidic dipeptidase-like 1	Naaladl1	0.28	7.45E-05
NLR family, apoptosis inhibitory protein 5	Naip5	0.33	1.57E-03
N-myc downstream regulated gene 1	Ndrg1	0.15	1.91E-06
nuclear factor, interleukin 3, regulated	Nfil3	0.37	3.10E-03
organic solute transporter beta	Ostb	0.22	5.40E-06
oxysterol binding protein-like 1A	Osbpl1a	0.21	2.60E-05
oxysterol binding protein-like 3	Osbpl3	0.47	6.02E-06
paired box gene 8	Pax8	0.34	4.77E-05

PDZ domain containing 3	Pdzd3	0.33	2.17E-07
phenazine biosynthesis-like protein domain containing	Pbld	0.34	6.81E-04
phosphatidylinositol-specific phospholipase C, X domain containing 2	Plcxd2	0.37	5.81E-06
phosphodiesterase 7A	Pde7a	0.49	9.99E-04
phospholipase B1	Plb1	0.27	4.88E-02
Phospholipid scramblase 1	Plscr1	0.45	2.23E-03
pleckstrin homology, Sec7 and coiled-coil domains, binding protein	Pscdbp	0.20	4.88E-06
predicted gene, EG226654	EG226654	0.42	4.34E-05
predicted gene, EG622976	EG622976	0.46	1.03E-03
prolactin receptor	Prlr	0.18	2.26E-08
pterin 4 alpha carbinolamine dehydratase/dimerization cofactor of hepatocyte nuclear factor 1 alpha (TCF1) 1	Pcbd1	0.46	8.23E-05
purine-nucleoside phosphorylase	Pnp	0.49	6.69E-05
pyruvate dehydrogenase kinase, isoenzyme 4	Pdk4	0.38	2.46E-03
Ral GEF with PH domain and SH3 binding motif 2	Ralgps2	0.49	1.30E-06
regenerating islet-derived 1	Reg1	0.22	6.05E-05
regucalcin	Rgn	0.12	2.37E-05
reticulon 4 receptor-like 1	Rtn4rl1	0.30	2.46E-05
retinol binding protein 2, cellular	Rbp2	0.49	4.19E-05
S100 calcium binding protein G	S100g	0.49	4.58E-05
sclerostin	Sost	0.21	3.74E-02
serine peptidase inhibitor, Kazal type 3	Spink3	0.06	1.90E-06
serum/glucocorticoid regulated kinase	Sgk	0.32	5.47E-03
serum/glucocorticoid regulated kinase 2	Sgk2	0.42	9.55E-04
short coiled-coil protein	Scoc	0.46	7.09E-04
similar to p47 protein	LOC547150	0.45	1.11E-04
small muscle protein, X-linked	Smpx	0.34	4.77E-04
solute carrier family 13 (sodium/sulphate symporters), member 1	Slc13a1	0.04	7.50E-07
solute carrier family 22 (organic cation transporter), member 1	Slc22a1	0.34	1.10E-06

solute carrier family 23 (nucleobase transporters), member 1	Slc23a1	0.09	2.80E-06
solute carrier family 25, member 36	Slc25a36	0.20	5.60E-07
solute carrier family 25, member 45	Slc25a45	0.04	2.99E-08
solute carrier family 26, member 3	Slc26a3	0.40	8.23E-06
solute carrier family 34 (sodium phosphate), member 2	Slc34a2	0.33	1.96E-02
solute carrier family 35, member A5	Slc35a5	0.41	3.64E-03
solute carrier family 37 (glucose-6-phosphate transporter), member 4	Slc37a4	0.29	3.90E-06
solute carrier family 39 (metal ion transporter), member 5	Slc39a5	0.48	1.82E-05
solute carrier family 41, member 2	Slc41a2	0.28	7.38E-06
solute carrier family 47, member 1	Slc47a1	0.30	1.93E-05
solute carrier family 5 (neutral amino acid transporters, system A), member 4b	Slc5a4b	0.06	1.52E-06
solute carrier family 5 (sodium/glucose cotransporter), member 12	Slc5a12	0.13	3.27E-03
solute carrier family 5 (sodium/glucose cotransporter), member 9	Slc5a9	0.07	4.73E-09
solute carrier family 5, member 4a	Slc5a4a	0.26	2.27E-04
solute carrier family 7 (cationic amino acid transporter, y+ system), member 9	Slc7a9	0.39	1.65E-04
solute carrier organic anion transporter family, member 2a1	Slco2a1	0.50	1.33E-04
somatostatin	Sst	0.43	3.55E-03
sorcin	Sri	0.46	1.51E-04
sphingomyelin synthase 2	Sgms2	0.45	1.52E-06
sphingosine-1-phosphate phosphatase 1	Sgpp1	0.49	1.88E-04
splA/ryanodine receptor domain and SOCS box containing 4	Spsb4	0.37	4.12E-04
sprouty homolog 3 (Drosophila)	Spry3	0.18	1.28E-05
SRY-box containing gene 13	Sox13	0.29	3.96E-05
SRY-box containing gene 6	Sox6	0.11	2.34E-06
sterol regulatory element binding factor 1	Srebf1	0.47	5.21E-03
succinate receptor 1	Sucnr1	0.31	3.54E-04
sulfotransferase family, cytosolic, 1C, member 2	Sult1c2	0.49	1.85E-02

synaptosomal-associated protein	Snap29	0.50	7.46E-06
TBC1 domain family, member 5	Tbc1d5	0.48	1.01E-04
tissue factor pathway inhibitor 2	Tfpi2	0.22	8.79E-07
transcription elongation factor A (SII), 3	Tcea3	0.27	2.74E-06
transmembrane 4 superfamily member 4	Tm4sf4	0.08	1.85E-06
transmembrane emp24 protein transport domain containing 6	Tmed6	0.03	1.29E-08
transmembrane protein 117	Tmem117	0.33	2.61E-03
transmembrane protein 140	Tmem140	0.41	3.67E-05
transmembrane protein 144	Tmem144	0.39	1.10E-06
transmembrane protein 37	Tmem37	0.48	1.49E-03
trimethyllysine hydroxylase, epsilon	Tmlhe	0.45	4.45E-05
troponin T2, cardiac	Tnnt2	0.46	1.36E-02
tubulointerstitial nephritis antigen	Tinag	0.02	6.11E-11
UDP glucuronosyltransferase 2 family, polypeptide A3	Ugt2a3	0.02	9.03E-09
UDP glucuronosyltransferase 2 family, polypeptide B34	Ugt2b34	0.29	2.45E-07
UDP glucuronosyltransferase 2 family, polypeptide B35	Ugt2b35	0.20	5.12E-04
UDP glucuronosyltransferase 2 family, polypeptide B36	Ugt2b36	0.04	3.22E-07
UDP glucuronosyltransferase 2 family, polypeptide B5	Ugt2b5	0.01	3.07E-07
ureidopropionase, beta	Upb1	0.35	6.00E-04
vanin 1	Vnn1	0.49	8.95E-04
von Willebrand factor A domain containing 1	Vwa1	0.11	5.89E-07
X transporter protein 3 similar 1 gene	Xtrp3s1	0.40	5.87E-06
yippee-like 2 (Drosophila)	Ypel2	0.41	5.66E-05
zinc finger with KRAB and SCAN domains 1	Zkscan1	0.22	1.28E-05
