# Supplementary material for: Loss of Hepatocyte-Nuclear-Factor-1α Impacts on Adult Mouse Intestinal Epithelial Cell Growth and Cell Lineages Differentiation
Source: PLoS One. 2010 Aug 24;5(8):e12378. doi: 10.1371/journal.pone.0012378 (PMC2927538; doi:10.1371/journal.pone.0012378)
Supplement: Table S2 — Genes annotated by IPA as involved in intestinal epithelial functions with altered expression in Hnf1α mutant jejunum samples as compared with control jejunum samples (N = 3). (0.03 MB PDF) [file pone.0012378.s002.pdf]

Gene	Gene Symbol	Fold change	P-value
**Molecular transport**			
aquaporin 1 (Colton blood group)	AQP1	-3.32	3.1E-06
aquaporin 7	AQP7	-3.49	9.4E-06
cytochrome P450, family 3, subfamily A, polypeptide 4	CYP3A4	-4.48	1.4E-03
gap junction protein, beta 1, 32kDa	GJB1	-2.37	9.1E-06
guanylate cyclase activator 2A (guanylin)	GUCA2A	-11.31	2.4E-05
solute carrier family 13 (sodium/sulfate symporters), member 1	SLC13A1	-24.76	7.5E-07
solute carrier family 22 (organic cation transporter), member 1	SLC22A1	-2.94	1.1E-06
solute carrier family 34 (sodium phosphate), member 2	SLC34A2	-3.02	2.0E-02
solute carrier family 47, member 1	SLC47A1	-3.35	1.9E-05
solute carrier organic anion transporter family, member 2A1	SLCO2A1	-2.09	1.3E-04
**Glucose metabolism and transport**			
glucose-6-phosphatase, catalytic subunit	G6PC	-3.10	1.7E-04
glucose-6-phosphate transporter	SLC37A4	-3.42	3.9E-06
lactase	LCT	-7.73	1.5E-04
pyruvate dehydrogenase kinase, isozyme 4	PDK4	-2.64	2.5E-03
**Lipid metabolism and transport**			
alpha-fetoprotein	AFP	-101.1	5.2E-09
apolipoprotein A-IV	APOA4	-2.32	1.3E-03
apolipoprotein C-II	APOC2	-4.22	3.1E-06
apolipoprotein L 7c	APOL7C	-2.32	9.5E-05
ATP-binding cassette, sub-family G (WHITE), member 8	ABCG8	-3.51	2.1E-06
fatty acid binding protein 1, liver	FABP1	-15.86	1.7E-07
**Enteroendocrine cells functions**			
chromogranin A (parathyroid secretory protein 1)	CHGA	-1.62	6.3E-03
chromogranin B (secretogranin 1)	CHGB	-1.99	1.7E-03
dipeptidyl-peptidase 4	DPP4	-2.86	9.2E-06
gastric inhibitory polypeptide	GIP	-1.83	5.5E-03
ghrelin/obestatin prepropeptide	GHRL	3,935	3,8E-05
neurotensin	NTS	-1.99	6,1E-04
peptide YY	PYY	-1.65	1.3E-03
somatostatin	SST	-2.34	3.6E-03

Supplemental Table 2.
Genes annotated by IPA as involved in intestinal epithelial functions with altered expression in *Hnf1α* mutant jejunum samples as compared with control jejunum samples (N=3).
