# Supplementary material for: Loss of Hepatocyte-Nuclear-Factor-1α Impacts on Adult Mouse Intestinal Epithelial Cell Growth and Cell Lineages Differentiation
Source: PLoS One. 2010 Aug 24;5(8):e12378. doi: 10.1371/journal.pone.0012378 (PMC2927538; doi:10.1371/journal.pone.0012378)
Supplement: Table S3 — Genes annotated by IPA as involved in protein translation control with modified expression in Hnf1α mutant jejunum samples as compared with control jejunum samples (N = 3). (0.03 MB PDF) [file pone.0012378.s003.pdf]

Genes annotated by IPA as involved in protein translation control with modified expression in *Hnf1α* mutant jejunum samples as compared with control jejunum samples (N=3).

Supplemental Table 3.

Gene	Gene Symbol	Fold Change	p-value
**Protein synthesis**			
eukaryotic translation initiation factor 2-alpha kinase 1	EIF2AK1	-1.62	7.59E-04
eukaryotic translation initiation factor 2B, subunit 1 alpha, 26kDa	EIF2B1	1.60	1.15E-04
eukaryotic translation initiation factor 2, subunit 3 gamma, 52kDa	EIF2S3	1.96	4.75E-02
glutamine-fructose-6-phosphate transaminase 1	GFPT1	1.85	1.28E-04
mitochondrial translational initiation factor 2	MTIF2	1.60	2.20E-02
myotubularin related protein 2	MTMR2	1.55	4.03E-04
ribonucleotide reductase M1	RRM1	1.53	7.17E-03
ribonucleotide reductase M2 polypeptide	RRM2	1.77	1.49E-03
transglutaminase 2	TGM2	1.84	1.61E-04
**Aminoacyl-tRNA biosynthesis**			
asparaginyl-tRNA synthetase	NARS	1.74	1.24E-03
cysteinyl-tRNA synthetase	CARS	1.64	4.39E-02
isoleucyl-tRNA synthetase	IARS	1.71	5.76E-03
isoleucyl-tRNA synthetase 2, mitochondrial	IARS2	1.75	4.49E-04
methionyl-tRNA synthetase	MARS	1.78	3.42E-03
tyrosyl-tRNA synthetase	YARS	1.58	2.17E-02
